# Familial Wolfram syndrome due to compound heterozygosity for two novel *WFS1* mutations

**Published:** 2008-07-25

**Authors:** Juan Carlos Zenteno, Gabriela Ruiz, Hector J. Pérez-Cano, Mayra Camargo

**Affiliations:** 1 Department of Genetics, “Conde de Valenciana,” Mexico City, Mexico; 2 Research Unit, “Conde de Valenciana,” Mexico City, Mexico; 3 Department of Neuroophthalmology, Institute of Ophthalmology “Conde de Valenciana,” Mexico City, Mexico

## Abstract

**Purpose:**

To describe the first instance of genotyping in a Latin American family with Wolfram syndrome (WS).

**Methods:**

Four affected siblings and their healthy parents were studied. Ophthalmologic examination included best corrected visual acuity determination, funduscopy, fluorescein retinal angiography, and Goldmann kinetic perimetry. Molecular methods included linkage analysis using microsatellites markers located on the markers located on the Wofram syndrome 1 (WFS1) region at 4p16.1, PCR amplification and direct nucleotide sequencing analysis of the complete coding region and exon/intron junctions of WFS1. In addition, allele-specific cloning and sequencing techniques were used to characterize a heterozygous frameshift mutation.

**Results:**

The four affected siblings presented with a homogeneous clinical picture characterized by early onset diabetes mellitus, severe optic atrophy, and progressive hearing loss. Linkage analysis indicated that all four sibs were heterozygous for markers linked to the *WFS1* region and that each inherited the same allele from the mother and the same from the father, suggesting compound heterozygosity. Direct *WFS1* analysis disclosed a paternally inherited novel missense R177P mutation whereas allele-specific cloning and sequencing revealed a novel *WFS1* 16 bp deletion that was inherited from the mother.

**Conclusions:**

Our report of two novel *WFS1* mutations expands the molecular spectrum of Wolfram syndrome. This is the first documented case of the molecular basis of the disease in a Latin American family. Analysis of more patients from this population will establish if compound heterozygosity is commonly found in affected individuals from this ethnic group.

## Introduction

Wolfram syndrome (WS; OMIM 222300) is an uncommon genetic disease defined by the association of early onset optic atrophy and diabetes mellitus, both appearing during the first two decades of life [1]. The disease is inherited as an autosomal recessive trait with an estimated prevalence of 1 in 770,000 and an estimated carrier frequency of 1 in 354; both figures are derived from a United Kingdom nationwide study of the syndrome [2]. WS is considered as a progressive neurodegenerative disorder that can include ataxia, peripheral neuropathy, urinary-tract atony, sensorineural hearing impairment, and psychiatric manifestations (reviewed in [3]]. WS is also known as DIDMOAD for diabetes insipidus, diabetes mellitus, optic atrophy, and deafness [4]. Brainstem atrophy frequently leads to central respiratory failure, which is the most common cause of death in WS individuals, usually during the fourth decade of life [2]. Other associated clinical findings in WS include gastrointestinal disorders, such as dismotility (constipation as well as chronic diarrhea), urinary incontinence, recurrent urinary infections, hidronephrosis, primary gonadal atrophy (males), and menstrual irregularities and delayed menarche [3,5]. Although there is considerable individual variation in clinical signs, the minimal diagnostic criteria are the concurrence of early onset (<15 years) diabetes mellitus, and progressive optic atrophy. These criteria give a positive predictive value for WS of 83% and a negative predictive value of 1% [2].

WS is caused by homozygous mutations in *WFS1*, a gene located at 4p16.1 and composed of seven coding exons [6,7]. *WFS1* encodes wolframin, an 890 amino acid glycoprotein that localizes primarily in the endoplasmic reticulum [8], where it has been shown to participate in the regulation of cellular calcium homeostasis [9]. To date, approximately 130 distinct mutations in *WFS1* have been identified in WS individuals from different ethnic backgrounds, and these include a variety of missense, nonsense, and frameshift insertion/deletion mutations [4,6,7,10-15]. Deleterious *WFS1* changes are distributed along the entire gene without apparent mutational hot spots.

The aim of this study is to describe the clinical and molecular features of an unusual WFS family composed by four affected sons of a non-consanguineous marriage. This is the first description of the molecular basis of WS in a Latin American family, and our results add to the genotypic spectrum of the disease.

## Methods

Four affected brothers from a nonconsanguineous Mexican family were included in the study. Both parents were healthy and had no familial history of optic atrophy, diabetes mellitus, hearing loss, or psychiatric disorders. Parents were nonconsanguineous and originated from distant regions of the country. This study was approved by the institutional review board and adhered to the tenets of the Declaration of Helsinki. Parents gave their informed consent for genetic analysis to be performed. Venous blood samples were drawn from both parents and their four sibs. Genomic DNA was immediately isolated from these samples using standard techniques.

### Case 1

**Figure 1:**
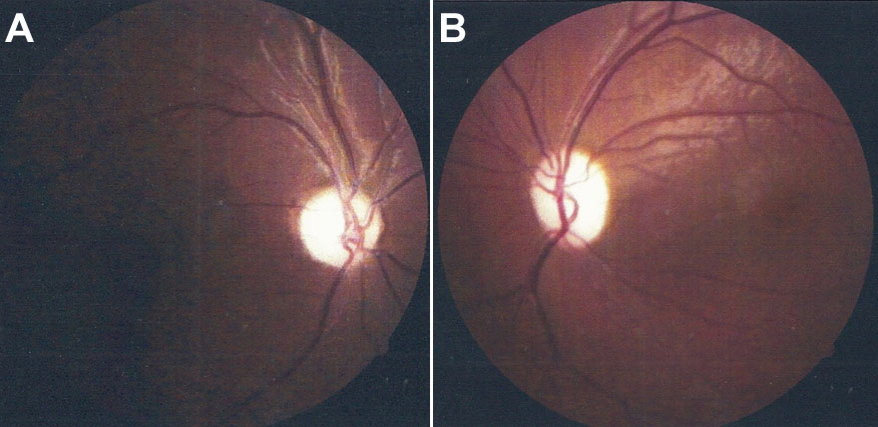
Funduscopic appearance of patients with Wolfram syndrome. Shown are fundus photographs from (**A**) 19-year-old case 1 (OD) and from (**B**) 14-year-old case 3 (OS). Both photographs reveal severe waxy pallor of the optic disc.

This was a 19-year-old male, the oldest of the four brothers, who was given a diagnosis of diabetes mellitus type 1 when he was 10 years old. He came to our hospital complaining of decreased vision. At examination, his visual acuity was 20/400 that improved after refraction to 20/300 in the right eye, and 3/200 that improved to 20/400 in the left eye. The pupils were mydriatic with decreased light reflex. Right optic nerve head showed a cup to disc ratio of 0.45 and severe pallor (Figure 1A). Microhemorrhages in the macular area were also evident; left optic nerve head had a cup to disc ratio of 0.25 and severe pallor. Goldmann Kinetic Perimetry (GKP) showed concentric reduction of 20 to 30 degrees mainly at the nasal sector of the visual field as well as an enlarged blind spot. Retinal fluorangiography (FAG) was normal in both eyes. Audiometric studies showed low-frequency hearing loss. Dental examination showed enamel hypoplasia and abnormal teeth color.

### Case 2

This was a 16-year-old male, who received a diagnosis of diabetes mellitus type 1 when he was 6 years old. He complained of having decreased vision for several years. On examination, his visual acuity was count fingers at 15 cm in the right eye that did not improve and hand movement in the left eye that did not improve. The pupils were mydriatic with decreased light reflex. Both optic nerve heads had a cup to disc ratio of 0.25 and exhibited severe pallor. GKP showed altitudinal arched vision islands with central vision not conserved in the right eye of 90x20 degrees and in the left eye of 60x10 degrees. FAG was normal in both eyes. Audiometric test showed low-frequency hearing loss. Dental examination showed enamel hypoplasia and abnormal coloration of teeth

### Case 3

This was a 14-year-old male, who was given a diagnosis of diabetes mellitus type 1 when he was 7 years old. He had a history of a surgical correction of a tracheoesophagic fistula at the age of 8 months. He presented with a history of decreased visual acuity. At ophthalmologic examination, his visual acuity was 20/100 in both eyes. Pupils were mydriatic with decreased light reflex. Optic nerve head cup to disc ratio was 0.25 in both eyes, and both optic discs showed severe pallor (Figure 1B). GKP and FAG were normal in both eyes. Audiometry showed low-frequency hearing loss. Dental examination showed enamel alterations and teeth discoloration.

### Case 4

The youngest brother was 11 years old. He received a diagnosis of diabetes mellitus type 1 when he was 5 years old. In the right eye, he had a visual acuity of 20/300 that improved to 20/200, and in the left eye, it was 20/400 that improved to 20/300. Pupils were mydriatic with decreased light reflex. Both optic nerve heads had a cup to disc ratio of 0.25 and exhibited severe pallor. In addition, pigmentary changes were observed in the macular area, bilaterally. GKP showed concentric reduction of 30 degrees and an enlarged blind spot. FAG was normal for both eyes. Audiometric tests revealed low-frequency hearing loss. Dental examination showed enamel alterations and changes in teeth coloration.

### Linkage analysis

DNA was isolated from peripheral blood leukocytes by standard procedures. Four microsatellite DNA markers (D4S432, D4S3023, D4S2366, and D4S2639) linked to the *WFS1* locus on 4p16.1 were used to determine linkage in the family [16]. Microsatellite analysis was performed in an ABI 310 Genetic Analyzer (Applied Biosystems, Foster City, CA) using the GenScan software (Applied Biosystems) for amplicon size determination.

### *WFS1* mutational analysis

The entire *WFS1* coding sequence (seven coding exons), as well as the intron/exon junctions, were amplified by PCR in DNA from the four affected patients and their parents using temperatures and primers available upon request. Each 25 μl PCR amplification reaction contained 1X buffer (20 μM Tris-Cl, 100 μM KCl, 0.1 μM EDTA, and 0.5% (v/v) Tween 20), 200 ng of genomic DNA, 0.2 mM of each dNTP, 2U *Taq* polymerase, 1 mM of forward and reverse primers (Table 1), and 1.5 mM MgCl_2_. PCR products were analyzed in 1.5% agarose gels from which the bands with the amplified templates were excised, and the DNA subsequently purified with the help of the Qiaex II kit (Qiagen, Hilden, Germany). Direct automated sequencing of *WFS1* was performed with the BigDye Terminator Cycle Sequencing kit (Applied Biosystems, Foster City, CA), adding about 15 ng of template DNA in each reaction and using a temperature program that included 25 cycles of denaturation at 97 °C for 30 s, annealing at 50 °C for 15 s, and extension at 60 °C for 4 min. Samples were analyzed in an ABI Prism 310 Genetic Analyzer (Applied Biosystems). Wild-type and mutated *WFS1* sequences were compared manually.

**Table 1:** - Oligonucleotide sequences used for PCR amplification and direct automated nuclotide sequencing of *WFS1* in this study.

**Exon**	**Primers (5’-3’)**	**Tm °c**
2	F: GCAGACACTAAGTGCCAGA	56
	R: CTGAACTGCAGAGGACCTG	
3	F: GCAGCAGCAGATCTGAAGA	56.6
	R: TCTCAGGCACCGACACTTCT	
4	F: GAAGTGGGTGAAAGGAGGT	53.9
	R: CAGTTAGCAAGCAGCATTAC	
5-6	F: GTCAGAGTGGCACCGAAAGC	65.1
	R: TCGCCCTGCAGGTGCAGGCTGGGA	
7	F: ATTGCTCTGTGTGAGGGTG	62.8
	R: CCTGCCTGAGGTGCGCGAGT	
8-1	F: TTGCCCAGAGGCAGGGTGGT	61.3
	R: ATGGAGGGCAGCAGCGATAGCA	
8-2	F: ATCCCCTGCTCGGAGCTGGCT	63
	R: AGTTGTAGACCTTCATGCC	
8-3	F: CAAGGCCAGCTTCTCTGTGGT	59.1
	R: ATGGCCTTGAGCTCGAAGACA	
8-4	F: CAAGGACATCGTGCTGCGGGC	63
	R: AGTCTGCACACGTGGGCACA	

Annealing temperature as are indicated at right. In this table (F) is forward primer, (R) is reverse primer.

### *WFS1* allele-specific cloning and sequencing

An allele-specific cloning and sequencing approach was used to precisely characterize a *WFS1* frameshift mutation. Briefly, new *WFS1* exon 8 products were amplified from genomic DNA, gel-purified, ligated by means of a TA-ligation method into the TA-cloning vector pGEM-T (Promega, Madison, WI), and subcloned into DH5α *E. coli* competent cells (Invitrogen, Carlsbad, CA) using standard procedures. The plasmid inserts were sequenced with the aforedescribed protocol and using the forward pUC/M13 primer. DNA from four independent clones was sequenced. Wild-type and mutated sequences were compared manually.

## Results

**Figure 2:**
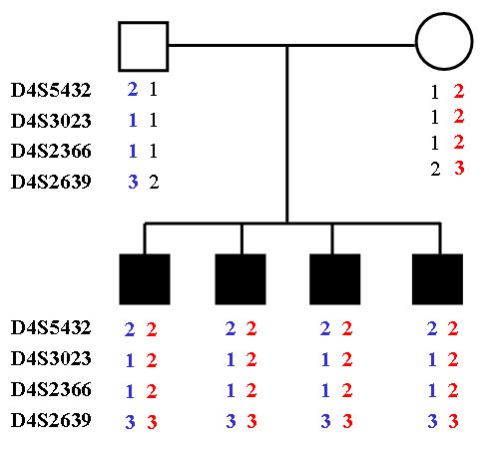
Linkage analysis for Wolfram syndrome locus markers at 4p16.1. Filled squares denote affected individuals. All four siblings inherited the same haplotype from their father (blue) and the same haplotype (red) from their mother, which suggested compound heterozygosity.

**Figure 3:**
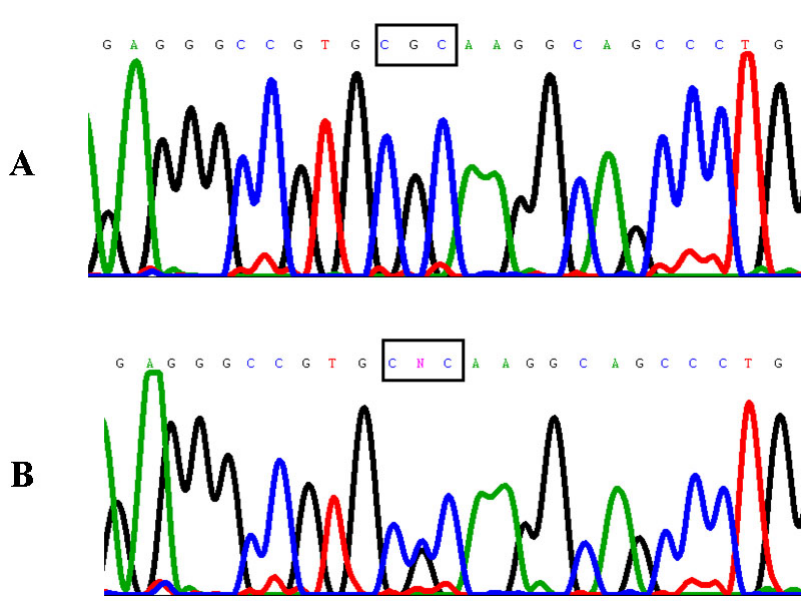
*WFS1* gene novel point mutation in WS. Exon 5 sequence analysis in DNA from an affected subject (case 1) revealed a heterozygous change from wild type guanine (**A**) to cytosine (**B**), predicting the change of arginine (CGC) to proline (CCC) in Wolframin residue 177.

**Figure 4:**
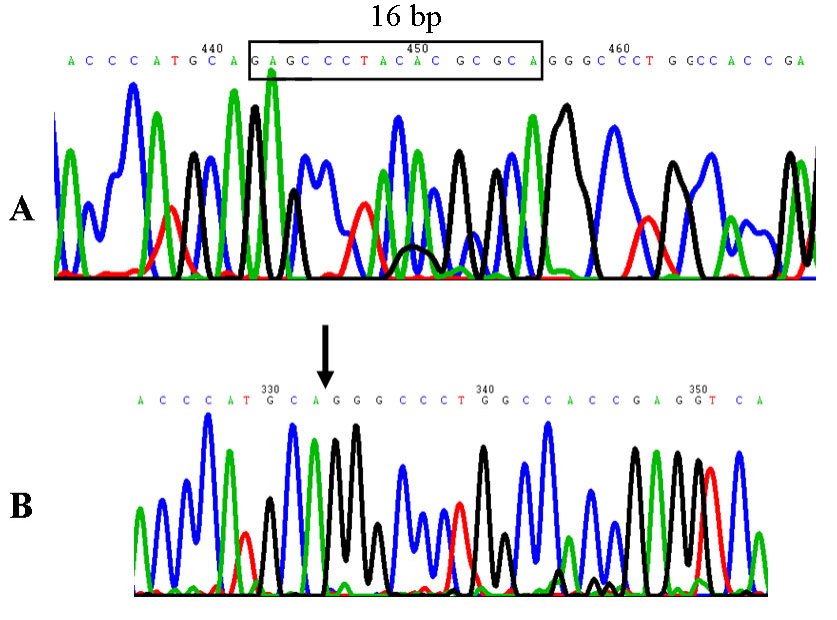
*WFS1* gene novel frameshift mutation in WS. Exon 8 sequence analysis revealed that a normal tract of 16 bp (boxed in A) is heterozygously deleted in DNA from an affected subject (case 1) (B). The deletion predicts the introduction of a premature stop signal 65 codons downstream residue 451 of Wolframin.

The clinical association of early onset diabetes mellitus and progressive optic atrophy suggested the diagnosis of WS in the four affected siblings from this family. As a first screening analysis, we performed linkage analysis to the *WFS1* locus at 4p16.1 by means of genotyping of markers D4S432, D4S3023, DS2366, and D4S2639. Visual inspection of the haplotypes revealed that all four affected siblings were heterozygous for the four markers as each parent consistently transmitted to their progeny the same *WFS1*-linked haplotype (Figure 2). These results strongly suggested *WFS1* compound heterozygosity as the source of the disease. Nucleotide sequencing of the entire *WFS1* coding region confirmed the presence of two different mutations in the four affected patients: one allele carried a novel R177P missense mutation resulting from a G>C transversion at nucleotide position 530 in exon 5 (Figure 3). This missense mutation was present heterozygously also in DNA from the father. Exon 8 DNA from the four affected siblings exhibited an overlapping pattern on automated DNA sequencing indicating the presence of a frameshift mutation. Allele-specific sequencing of a fragment of exon 8 allowed the identification of a novel 16 bp deletion, 1355–1370delAGC CCT ACA CGC GCA G (c.1354del16), that predicts the introduction of a premature stop signal 65 codons downstream, P451fsX515 (Figure 4). Maternal DNA analysis demonstrated that the mother carried this 16 bp deletion in a heterozygous state.

## Discussion

WS is an autosomal recessive disorder characterized by the association of juvenile-onset diabetes mellitus and optic atrophy. The disease is caused by homozygous mutations in the gene *WFS1* at 4p16.1. A second form of WS, *WFS2*, maps to 4q22-q25 [16] and has been recently demonstrated to be caused by homozygous mutations in a highly conserved zinc-finger gene, *ZCD2* [17]. Similar to Wolframin, ERIS, the *ZCD2* encoded protein, also localizes to the endoplasmic reticulum [17].

Molecular defects in *WS1* are associated with non-WS phenotypes. Heterozygous mutations in *WFS1* have been demonstrated to be a cause of dominantly inherited nonsyndromic low-frequency sensorineural hearing loss [18]. Moreover, an association between *WFS1* single nucleotide polymorphisms and an increased risk of type 2 diabetes has been identified in European populations [19,20]. This is supported by the previous observation of an increased prevalence of diabetes mellitus in first-degree relatives of WS patients [21]. Similarly, heterozygous carriers of *WFS1* mutations have an increased incidence of psychiatric disorders including endogenous depression, suicide attempts, short-term memory loss, and anxiety [22].

In this work we have described the clinical and molecular features of four Mexican siblings who have WS. To the best of our knowledge, this is the first Latin American WS family in which the molecular basis of the disease has been analyzed. This family was unusual as all the four descendants of a non-consanguineous couple were affected. The parents were not relatives, originated from distant geographic areas of the country, and each of them was a heterozygous carrier for a different *WFS1* mutation. WFS prevalence or carrier frequencies in Mexico are currently unknown. A carrier frequency of 1 in 354 was estimated by Barret et al. [2]. in the United Kingdom.

Although compound heterozygosity for WFS has been previously reported [14,15,23], the present pedigree is an unusual compound heterozygosity familial example as the four sons of this unrelated couple inherited a mutated allele from each parent and were affected. This greatly deviates from the empiric 25% risk of autosomal recessive diseases.

Genetic analysis revealed that affected members of this family were compound heterozygous for two novel *WFS1* mutations, including a paternally inherited missense mutation at exon 5 and a maternally inherited 16 bp deletion at exon 8. The missense mutation at exon 5 replaces an arginine residue at position 177 in the Wolframin extracellular 1 domain, while the 16 bp deletion predicts a frameshift from amino acid 451, in the transmembrane 4 domain, and the introduction of a premature stop codon 65 residues downstream. An arginine residue at position 177 is evolutionarily conserved among Wolframin proteins in several species, indicating its biologic significance.

Besides the typical features of WS, all four patients exhibited a homogeneous pattern of dental anomalies characterized by enamel hyoplasia and abnormalities in teeth color. To the best of our knowledge, dental anomalies have not been reported as being associated with WS and it is unknown to us if dental anomalies in this family were related to the specific *WFS1* compound mutation.

In conclusion, our study analyzes for the first time the *WFS1* gene status in a Latin American family with WS and adds to the mutational spectrum of the disease. Molecular investigation of more samples of WS patients from our country will help to identify if compound heterozygosity occurs commonly or if there are recurrent or founder *WFS1* mutations in this particular ethnic group.
